# Heme Oxygenase-1 Induction in Human BeWo Trophoblast Cells Decreases *Toxoplasma gondii* Proliferation in Association With the Upregulation of p38 MAPK Phosphorylation and IL-6 Production

**DOI:** 10.3389/fmicb.2021.659028

**Published:** 2021-04-12

**Authors:** Marcos Paulo Oliveira Almeida, Caroline Martins Mota, Tiago Wilson Patriarca Mineo, Eloisa Amália Vieira Ferro, Bellisa Freitas Barbosa, Neide Maria Silva

**Affiliations:** ^1^Laboratory of Immunopathology, Institute of Biomedical Sciences, Federal University of Uberlândia, Uberlândia, Brazil; ^2^Laboratory of Immunoparasitology “Dr. Mário Endsfeldz Camargo,” Institute of Biomedical Sciences, Federal University of Uberlândia, Uberlândia, Brazil; ^3^Laboratory of Immunophysiology of Reproduction, Institute of Biomedical Sciences, Federal University of Uberlândia, Uberlândia, Brazil

**Keywords:** BeWo villous trophoblast cells, HTR-8/SVneo extravillous trophoblast cells, heme oxygenase-1, interleukin-6, p38 MAPK, *Toxoplasma gondii*

## Abstract

Heme oxygenase-1 (HO-1) enzyme exerts beneficial effects at the maternal-fetal interface, especially in trophoblasts, being involved in survival and maturation of these cell phenotypes. Trophoblast cells play essential roles throughout pregnancy, being the gateway for pathogens vertically transmitted, such as *Toxoplasma gondii*. It was previously shown that HO-1 activity was involved in the control of *T. gondii* infection *in vivo*; however, its contribution in trophoblast cells during *T. gondii* infection, remain undefined. Thus, this study aimed to investigate the influence of HO-1 in *T. gondii*-infected BeWo and HTR-8/SVneo human trophoblast cells. For this purpose, trophoblast cells were infected and the HO-1 expression was evaluated. *T. gondii*-infected BeWo cells were treated with hemin or CoPPIX, as inducers of HO-1, or with bilirubin, an end-product of HO-1, and the parasitism was quantified. The involvement of p38 MAPK, a regulator of HO-1, and the cytokine production, were also evaluated. It was found that *T. gondii* decreased the HO-1 expression in BeWo but not in HTR-8/SVneo cells. When treated with the HO-1 inducers or bilirubin, BeWo cells reduced the parasite proliferation. *T. gondii* also decreased the p38 MAPK phosphorylation in BeWo cells; on the other hand, HO-1 induction sustained its activation. Finally, the IL-6 production was upregulated by HO-1 induction in *T. gondii*-infected cells, which was associated with the control of infection.

## Introduction

The placenta is a transient organ formed during human pregnancy, responsible for establishing an important maternal-fetal communication which performs metabolic, nutritional, endocrine, and immunological functions, favoring a successful embryonic development (reviewed by Burton and Fowden, 2015). Placenta, the unique exchange organ between mother and fetus is derived from the extraembryonic tissues. After implantation of blastocyst, trophectoderm generates the first trophoblast lineages, early mononuclear cytotrophoblasts and the multinuclear primitive syncytium that quickly invades the maternal tissues. The trophoblast cells proliferate and form the chorionic villi that present a mesenchymal compartment rouse from extraembryonic mesoderm. The normal placentation occurs by the direct contact of trophoblast cells with the maternal decidua, which comprises the developed endometrium, constituted by endometrial glands, uterine blood vessels, decidual stromal cells, and immune cells. The interaction between these fetal and maternal elements constitutes the maternal-fetal interface (reviewed by Knöfler et al., 2019; Turco and Moffett, 2019). Human trophoblast cells at the initial stage, also known as cytotrophoblastic stem cells, differentiate in two specific cell lineages, the villous and extravillous trophoblast (reviewed by Gude et al., 2004). Villous trophoblast are non-migratory, highly proliferative and fusogenic cells that originate an additional cell component, the syncytiotrophoblast. Together, these constitute the chorionic villi, structures that anchor to the decidua. Moreover, the villous trophoblast develops migratory and invasive properties differentiating in extravillous trophoblast, a decidual remodeling cell population that favors a healthy placentation (Kurman et al., 1984; reviewed by Graham and Lala, 1992; Gude et al., 2004). Trophoblast cells produce essential mediators, such as hormones and cytokines, which leads to adaptations in maternal immune cells establishing a cooperative status that prevents fetal rejection (reviewed by Mor et al., 2011). In addition, one of the key cellular elements that contribute with the maternal-fetal tolerance and placental physiology is the heme oxygenase (HO) enzymatic system (reviewed by Levytska et al., 2013).

The HO microsomal enzyme is associated with oxidative stress conditions in various cell types, being responsible to catalyze the degradation of free heme, leading to formation of three end-products: iron, that binds to ferritin for storage in the cells, gas carbon monoxide (CO) and biliverdin, which is consecutively converted to bilirubin by biliverdin reductase (BVR) (Tenhunen et al., 1968, 1969; reviewed by Soares and Bach, 2009). To date, three HO isoforms, HO-1, HO-2, and HO-3 were identified and HO-1 is the only inducible isoform, codified by the *HMOX1* gene and activated by a broad range of chemical and physical stimuli. HO-1 is the most relevant isoenzyme, since its activity favors cellular protection pathways, triggering antioxidant, anti-inflammatory, and antiapoptotic effects. Different stimuli such as hypoxia, radiation, hormones, growth factors, cytokines, and heme itself, lead to HO-1 expression by activation of cell signaling pathways, that includes the mitogen-activated protein kinases (MAPK) (reviewed by Ryter et al., 2006; Soares and Bach, 2009). The MAPK superfamily comprises three major serine/threonine kinases: the p38 MAPK, the extracellular signal-regulated kinase (ERK), and the c-Jun N-terminal kinase (JNK) (reviewed by Ryter et al., 2006). In broad context, the stimuli that induce HO-1 initially recruit p38 MAPK cascade, which leads the activation of specific transcription factors for *HMOX1* expression. Also, the own HO-1 activity may activate p38 MAPK, resulting in beneficial effects that characterize this pathway as a major regulator of HO-1 (reviewed by Ryter and Otterbein, 2004; Ryter et al., 2006; reviewed by Paine et al., 2010).

Previously, the HO-1 expression at the human maternal-fetal interface was demonstrated, being detected in trophoblast cells that include villous and extravillous trophoblasts (Lyall et al., 2000; Yoshiki et al., 2000). HO-1 modulates directly survival and maturation of trophoblast cells and promotes the fetal tolerance (reviewed by Schumacher and Zenclussen, 2015); however, the mechanisms that induce immunological tolerance can increase the susceptibility to infections caused by several types of pathogens, such as *Toxoplasma gondii*, which infects trophoblast cells and may be vertically transmitted to the embryo/fetus (reviewed by Arora et al., 2017).

*Toxoplasma gondii* replicates inside of different nucleated cell types and spreading through host tissues, leading to the clinical manifestations more adverse in immunocompromised individuals and during pregnancy (reviewed by Robert-Gangneux and Dardé, 2012). Depending on gestational age, *T. gondii* reaches the maternal-fetal interface and gradually transposes the placental barrier toward embryo/fetus, characterizing the congenital toxoplasmosis that leads to malformations and neurological disorders with visual injuries in neonates, or even miscarriage in severe cases (reviewed by Heerema-McKenney, 2018). *T. gondii* triggers in the host a cellular immune response characterized by a Th1 profile with increased pro-inflammatory cytokines, such as interleukin (IL)-12, interferon (IFN)-γ, tumor necrosis factor (TNF), IL-6, in parallel with IL-10 production (reviewed Denkers and Gazzinelli, 1998). In BeWo trophoblast cells, the infection induces macrophage migration inhibitory factor (MIF), IL-12 and IL-6 production (Castro et al., 2013), and in HTR-8/SVneo trophoblast cells, *T. gondii* infection also increases the IL-6 production (Guirelli et al., 2015). Because a Th1-biased immune response controls *T. gondii* growth and, the Th2/T regulatory profiles favors a normal pregnancy, a balance between these responses is essential to promote fetal tolerance (reviewed by Borges et al., 2018).

Previously, our group investigated the role of HO-1 in *T. gondii* experimental infection *in vivo*, and we showed that the HO-1 activity was involved in the control of *T. gondii* replication in the lung of resistant BALB/c mice, and also in this organ and in the small intestine of susceptible C57BL/6 mice (Araujo et al., 2013). Additionally, the protective function of HO-1 during bacterial pathogenesis in murine pregnancy was demonstrated, since the infections with *Brucella abortus* or *Listeria monocytogenes* reduced the HO-1 expression in the placenta and in the trophoblast giant cells, which was associated with increased cell death in these trophoblasts and greater abortion rates in infected pregnant mice (Tachibana et al., 2008, 2011).

BeWo, a choriocarcinoma cell line (Pattillo and Gey, 1968), has morphological and functional properties common to normal villous trophoblast, since it produces placental hormones (Jeschke et al., 2007), expresses epithelial markers (Abou-Kheir et al., 2017), and also the HO-1 enzyme (Bilban et al., 2009). Additionally, BeWo cells express IL-6, IL-10, IFN-α, IFN-β, and granulocyte macrophage colony stimulating factor (GM-CSF) mRNA expression as do normal human trophoblast obtained from term placenta (Bennett et al., 1996); IL-10 (Bennett et al., 1997) and human chorionic gonadotropin production that represents a fundamental property of the trophoblast (Pattillo and Gey, 1968). Likewise to BeWo, JAR choriocarcinoma is another established human placental cell line that has also a phenotype of villous trophoblast, and both are used as trophoblasts model for *in vitro* studies (Apps et al., 2009). However, the BeWo cell line is the most commonly used cell model to mimic the human villous trophoblast population *in vitro* (reviewed by Abou-Kheir et al., 2017). HTR-8/SVneo extravillous trophoblast cells are non-tumorigenic and non-metastatic cell line, synthesize human chorionic gonadotropin (hCG), and express markers of epithelial-mesenchymal transition (EMT) (Graham et al., 1993; Msheik et al., 2020). Specifically, in the present study, BeWo and HTR-8/SVneo cells were used, as several studies have been done related to the interaction of these cell phenotypes with *T. gondii* (Oliveira et al., 2006; Castro et al., 2013; Barbosa et al., 2014, 2015; Guirelli et al., 2015; Almeida et al., 2019; Milian et al., 2019). Thus, these cell lines are promising models to investigate pathophysiology of *T. gondii* infection in trophoblast (Barbosa et al., 2015; Guirelli et al., 2015; Almeida et al., 2019).

Considering the relevance of HO-1 in trophoblast physiology, and that its induction was able to decrease *T. gondii* growth in mice, the present study aimed to investigate the role of HO-1 in the infection of BeWo villous and HTR-8/SVneo extravillous trophoblast cells. For this purpose, firstly, we verified the HO-1 expression levels in these two kinds of human trophoblast cells under *T. gondii* infection. As the infection decreased the HO-1 expression levels in BeWo but did not interfere in its expression in HTR-8/SVneo cells, in the additional experiments, the effect of HO-1 induction was investigated on *T. gondii* proliferation, p38 MAPK phosphorylation, and IL-6, MIF, and TNF production during the infection with the parasite in BeWo cells.

## Materials and Methods

### Culture of BeWo and HTR-8/SVneo Cells

The BeWo human choriocarcinoma cell line was obtained from American Type Culture Collection (CCL-98^TM^, ATCC^®^, Manassas, VA, United States), and the HTR-8/SVneo human immortalized extravillous trophoblast cell line was kindly provided by Dr. Estela Bevilacqua (University of São Paulo, SP, Brazil). Both cell lines were cultured in 25 or 75 cm^2^ culture flasks, in Roswell Park Memorial Institute (RPMI)-1640 medium (Cultilab, Campinas, SP, Brazil) supplemented with 2 mM L-glutamine, 100 U/ml penicillin, 100 μg/ml streptomycin (Sigma-Aldrich, St. Louis, MO, United States), and 10% heat-inactivated fetal bovine serum (FBS) (Cultilab) (complete medium), in humidified incubator at 37°C and 5% CO_2_ (Almeida et al., 2019). According to protocol number 13/2012, the Ethics Committee of the Federal University of Uberlândia, MG, Brazil, determines that the commercially acquired cell lines do not require ethical approval.

### Parasites

*Toxoplasma gondii* tachyzoites of 2F1 clone, a highly virulent (RH) strain, which constitutively expresses the cytoplasmic beta-galactosidase enzyme (β-gal) (Seeber and Boothroyd, 1996), were kindly provided by Dr. Vern B. Carruthers (Medical School of Michigan University, United States) and maintained by passage every 2 days in monolayers of HeLa human cervix adenocarcinoma cell line (acquired from ATCC), cultured in RPMI medium supplemented with 2% FBS (Almeida et al., 2019). In all procedures of experimental infection, confluent culture flasks of *T. gondii*-infected HeLa cells were scraped and cell suspension passed three times through 21-gauge needle, and then in 26-gauge needle. Next, free tachyzoites were separated from host cells by low-speed centrifugation (70 × *g*, 3 min) to eliminate cell debris, parasites were collected, centrifuged again (720 × *g*, 5 min), counted, and used in subsequent assays.

### Chemical Induction of HO-1

To investigate the effects of HO-1 induction in trophoblast cells, the porphyrin hemin (Sigma-Aldrich), a synthetic iron protoporphyrin-IX or the cobalt protoporphyrin-IX (CoPPIX) (Frontier Scientific, Logan, UT, United States), were employed as HO-1 chemical inducers (Tenhunen et al., 1969; reviewed by Ryter et al., 2006). For the experiments, hemin or CoPPIX (10 mM) were reconstituted, at the time of use, in sodium hydroxide at 10% (NaOH 100 mM) in RPMI medium without FBS. Then, the treatments in the specific experimental doses were prepared in RPMI-complete medium. In parallel, cells were also incubated only with NaOH in the absence of hemin or CoPPIX, as vehicle condition, or only with RPMI-complete medium (control). Additionally, all solutions were manipulated under light protection, avoiding formation of free radicals and consequent loss of the porphyrins biological activity (Vasconcellos et al., 2016).

### Viability of BeWo Cells Treated With HO-1 Inducers

In order to evaluate the effects of hemin or CoPPIX treatments on viability of BeWo cells, the MTT [3-(4,5-dimethylthiazol-2-yl)-2,5-diphenyltetrazolium bromide] assay was performed, as previously described (Mosmann, 1983). For this purpose, BeWo (3 × 10^4^/200 μl/well) cells were cultured in 96-well plate in complete medium for 24 h at 37°C and 5% CO_2_. Next, cells were treated with hemin (Liu et al., 2016) or CoPPIX (10, 40, 80, 100, or 200 μM) or incubated with vehicle (0.2% NaOH) or with only complete medium (control), for an additional 24 h. In a parallel assay, BeWo (3 × 10^4^/200 μl/well) cells were cultured in 96-well plate, for 24 h, and treated with exogenous bilirubin (1, 10, 40, 80, or 100 μM) (Cayman Chemical, Ann Arbor, MI, United States), as the end-product of the HO-1 activity, or incubated with 0.2% dimethyl sulfoxide (DMSO), as vehicle, or with complete medium (control), during 24 h. At the end, the supernatants of both experiments were discarded and the cells were incubated with 10 μl of MTT reagent (Sigma-Aldrich) diluted in sterile phosphate-buffered saline (PBS) at 5 mg/ml plus 90 μl of complete medium, for 4 h at 37°C and 5% CO_2_. After, supernatants were removed and the cells were homogenized at room temperature with 100 μl of 10% sodium dodecyl sulfate (SDS) and 50% dimethylformamide (Sigma-Aldrich) to completely dissolve the formazan crystal. The absorbance was measured in a microplate reader (Versa Max ELISA Microplate Reader, Molecular Devices, Sunnyvale, CA, United States) at 570 nm, and the data were expressed as the percentage of viable cells in relation to the control (medium as untreated cells—100% viability).

### Quantification of *T. gondii* Proliferation in BeWo Cells

In order to investigate the effect of hemin or CoPPIX treatments (HO-1 induction) on *T. gondii* intracellular proliferation, the β-galactosidase assay was performed. Firstly, BeWo (3 × 10^4^/200 μl/well) cells were cultured in 96-well plates in complete medium for 24 h at 37°C and 5% CO_2_. Then, the cells were infected with *T. gondii* at proportion of one parasite per cell (1:1), in medium with 2% FBS and incubated for 3 h. Next, the cells were washed with incomplete medium (without FBS) to remove non-internalized parasites and treated with hemin or CoPPIX (10, 40, or 80 μM) or incubated with vehicle (0.08% NaOH), as a control of untreated cells, for additional 24 h. After, the cell-free supernatants were collected and stored at −80°C for posterior cytokine measurement. Secondly, BeWo (3 × 10^4^/200 μl/well) cells were cultured in 96-well plate, for 24 h, infected with *T. gondii* (1:1) in medium with 2% FBS, incubated for 3 h, washed with incomplete medium, and treated with exogenous bilirubin (1, 10, 40, 80, or 100 μM) or incubated with vehicle (0.2% DMSO) during 24 h. Finally, in a third set of experiments, the impact of the host p38 MAPK inhibition was verified on *T. gondii* intracellular proliferation in the context of this study. For this purpose, BeWo (3 × 10^4^/200 μl/well) cells were cultured again in 96-well plate, during 24 h, and pretreated for 3 h with 10 μM of SB203580 (Sigma-Aldrich), a specific p38 MAPK inhibitor (Kweider et al., 2011), or incubated only with medium as a control of untreated cells. Next, the supernatants were discarded, the cells washed with incomplete medium to remove any remnant of this MAPK inhibitor (Barbosa et al., 2014), and subsequently infected with *T. gondii* (1:1) in medium with 2% FBS, for an additional 3 h. After, the cells were washed again to remove non-internalized tachyzoites and treated with hemin (80 μM) or incubated only with complete medium (control) during 24 h. At the end, for the three experiments described above, the parasite quantification on cell samples was performed by the β-galactosidase reaction using the chlorophenol red-β-D-galactopyranoside reagent substrate (CPRG; Roche Diagnostics, Mannheim, Germany) (Almeida et al., 2019). The absorbance was measured in microplate reader at 570 nm, and the *T. gondii* intracellular proliferation ratio was calculated in relation to a standard curve of 2F1 tachyzoites, ranging from 1 × 10^6^ to 15.625 × 10^3^ total parasites. The data were expressed as number of tachyzoites (× 10^3^) in all conditions carried out. When appropriated, the data obtained from this assay were used to calculate the inhibition rates of *T. gondii* proliferation, expressed as percentage (%) of the control (vehicle).

### Western Blotting for HO-1 and Phosphorylated p38 MAPK

In order to analyze the HO-1 protein expression and phosphorylated p38 MAPK, western blotting assays were performed. Firstly, BeWo (1 × 10^6^/2,000 μl/well) and HTR-8/SVneo (5 × 10^5^/2,000 μl/well) cells were cultured in six-well plates in complete medium for 24 h at 37°C and 5% CO_2_. Since the extravillous cytotrophoblast cells are larger than villous cytotrophoblastic cells (Kurman et al., 1984), 5 × 10^5^/well of HTR-8/SVneo cells was used to obtain a similar cellular growth in 24 h incubation, as do BeWo cells. Next, the cells were infected with *T. gondii* (1:1), or not as a control of non-infected cells, in medium with 2% FBS and incubated for 3 h. Then, the cells were washed with incomplete medium to remove non-internalized parasites and cultured with a new complete medium, during an additional 24 h. In a second set of experiments, BeWo (1 × 10^6^/2,000 μl/well) cells were cultured in six-well plates in complete medium for 24 h, infected, or not, with *T. gondii* (1:1) in medium with 2% FBS for 3 h, washed with incomplete medium, and treated with hemin (80 μM) or with vehicle (0.08% NaOH) in complete medium, for an additional 24 h. At the end, the cells were harvested, centrifuged, homogenized, and lysed with radioimmunoprecipitation (RIPA) lysis buffer (50 mM Tris-HCl, 150 mM sodium chloride (NaCl), 1% (*v*/*v*) Triton X-100, 1% (*w*/*v*) sodium deoxycholate, 0.1% (*w*/*v*) sodium dodecyl sulfate (SDS), and pH 7.5), supplemented with protease inhibitor cocktail (Complete^®^ Lysis-M EDTA-free; Roche Diagnostics, Mannheim, Germany) and phosphatase inhibitors (1 mM sodium orthovanadate (Na_3_VO_4_) and 25 mM sodium fluoride (NaF)), and submitted to four freeze-thaw cycles, in liquid nitrogen and heated water (37°C), respectively. The protein concentration of the samples was measured by the Bradford method (Bradford, 1976). Then, samples (80 μg) were submitted to electrophoresis in polyacrylamide gel (SDS-PAGE at 12%), under reducing conditions, and electrotransferred to polyvinylidene fluoride (PVDF) membranes (Immobilon^®^-FL, Millipore Corporation, Billerica, United States). The membranes were stained with 0.5% Ponceau (Sigma-Aldrich) to confirm that proteins were equally loaded. Then, the membranes were blocked for 1 h with blotting buffer (25 mM Tris-HCl, 150 mM NaCl, 0.1% (*v*/*v*) Tween 20, and pH 7.4) added with 4% non-fat dry milk (Molico, Nestlé^®^, São Paulo, SP, Brazil) and incubated, overnight, with the following primary antibodies diluted in a blotting buffer with 2% non-fat dry milk: goat polyclonal anti-human-HO-1 (1:1,000; Santa Cruz Biotechnology Cat# sc-1797, Santa Cruz, CA, United States), rabbit polyclonal anti-human-phospho-p38 MAPK (T^180^/Y^182^) (1:400; R&D Systems Cat# AF869, Minneapolis, MN, United States), or mouse monoclonal anti-human-β-actin (1:1,000; Santa Cruz Biotechnology Cat# sc-81178). Next, the membranes were washed and incubated, for 2 h, with the respective horseradish-peroxidase secondary antibodies, diluted in a blotting buffer with 2% non-fat dry milk: rabbit anti-goat-HRP (1:3,000; Jackson ImmunoResearch Cat# 305-035-003, Newmarket, Suffolk, United Kingdom), goat anti-rabbit-HRP (1:1,000; R&D Systems Cat# HAF008; or Sigma-Aldrich Cat# A9169), or goat anti-mouse-HRP (1:3000; Sigma-Aldrich Cat# A3673). Finally, membranes were washed again and the reactions were developed using an enhanced chemiluminescent substrate (ECL) (SuperSignal^®^ West Pico kit, Thermo Fisher Scientific, United States). Photodocumentation of the membranes was obtained on translluminator ChemiDoc^TM^ MP Imaging System (Bio-Rad Laboratories, Hercules, CA, United States), and densitometric analysis was performed by Image Lab^TM^ Software (Bio-Rad Laboratories). Data were expressed as relative expression of the interest protein (HO-1 or phospho-p38 MAPK) in relation to endogenous protein control (β-actin).

### Measurement of Cytokines Secreted by BeWo Cells

The detection of human cytokines secreted by BeWo cells under *T. gondii* infection and/or hemin treatment (HO-1 induction) was carried out by sandwich enzyme-linked immunosorbent assay (ELISA). The levels of IL-6, TNF (both OptEIA^TM^, BD Bioscience, San Diego, CA, United States), and MIF (DuoSet^®^, R&D Systems, Minneapolis, MN, United States) were measured in the cell supernatants following the manufacturer’s instructions. Supernatants from non-infected and/or untreated cells were used as control. The detection limits for IL-6, TNF, and MIF were 4.7, 7.8, and 31.25 pg/ml, respectively.

### Statistical Analysis

The data obtained were expressed as mean and standard error of the mean (± SEM), of up to three independent experiments carried out in six to eight replicates. Differences between two groups were assessed by unpaired Student’s *t* test or by Mann-Whitney test, for parametric or non-parametric data, respectively. When appropriate, differences among multiple groups were assessed by one-way analysis of variance (ANOVA) test with Bonferroni’s multiple comparison posttest, for parametric data, or by Kruskal-Wallis test with Dunn’s multiple comparison posttest, for non-parametric data. Statistical analysis was performed using GraphPad Prism version 7.0 (GraphPad Software, Inc., San Diego, CA, United States), and the differences were considered statistically significant when *P* < 0.05.

## Results

### *T. gondii* Infection Decreases the HO-1 Expression in BeWo Villous but Not in HTR-8/SVneo Extravillous Trophoblast Cells

**FIGURE 1:**
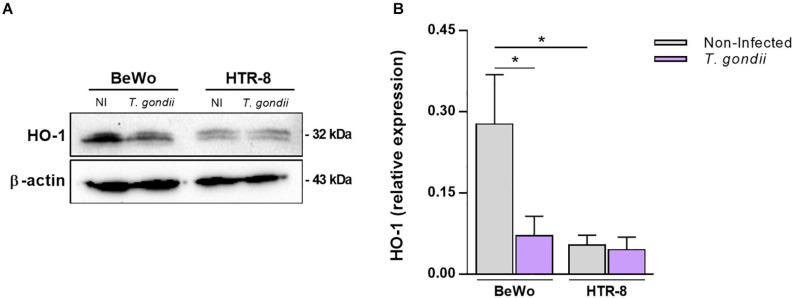
Influence of *Toxoplasma gondii* infection in the HO-1 expression by BeWo and HTR-8/SVneo trophoblast cells. BeWo (1 × 10^6^ cells/2,000 μl/well) and HTR-8/SVneo (5 × 10^5^ cells/2,000 μl/well) cells were cultured in six-well plate during 24 h, infected or not (control) with *T. gondii* (1:1) for 3 h, and incubated with complete medium for additional 24 h. Cells were lysed, the total protein content were separated by electrophoresis, transferred to PVDF membranes, and submitted to western blotting assays for detection of HO-1 and β-actin. Protein bands were photodocumented, and densitometric analysis was performed by the ratio among HO-1 and its corresponding β-actin (endogenous control). **(A)** Representative blots for HO-1 and β-actin in the non-infected (NI) or infected (*T. gondii*) cell lines (cited as BeWo or HTR-8). **(B)** Densitometric analysis for HO-1 expressed as mean ± SEM from two independent experiments performed in six replicates. Significant differences were statistically assessed by the Mann-Whitney test; *P < 0.05.

It was initially investigated whether infection by *T. gondii* would influence HO-1 expression in BeWo and HTR-8/SVneo trophoblast cells after 24 h of exposure to the parasite. Our data obtained from western blotting assays revealed that HO-1 expression was significantly reduced in *T. gondii*-infected BeWo cells in comparison with the control non-infected cells (*P* = 0.0226), as observed in the representative protein bands (Figure 1A) and densitometric analysis (Figure 1B). Regarding the HTR-8/SVneo cells, the infection did not alter the enzyme expression, since the levels of HO-1 were similar to those detected in non-infected HTR-8/SVneo cells (Figures 1A,B). Furthermore, in the absence of *T. gondii*, the HO-1 expression in normal conditions was significantly higher in BeWo cells than in HTR-8/SVneo (*P* = 0.0286), suggesting that BeWo villous trophoblast cells present more amounts of HO-1 than HTR-8/SVneo extravillous trophoblasts. Thus, because in BeWo cells the HO-1 expression was more pronounced, and the *T. gondii* infection induced a significant downregulation in the HO-1 levels, we performed the following experiments using this cell lineage as model of trophoblast cells.

### The Increase of HO-1 Expression by Hemin Treatment Decreases the *T. gondii* Proliferation in BeWo Trophoblast Cells

**FIGURE 2:**
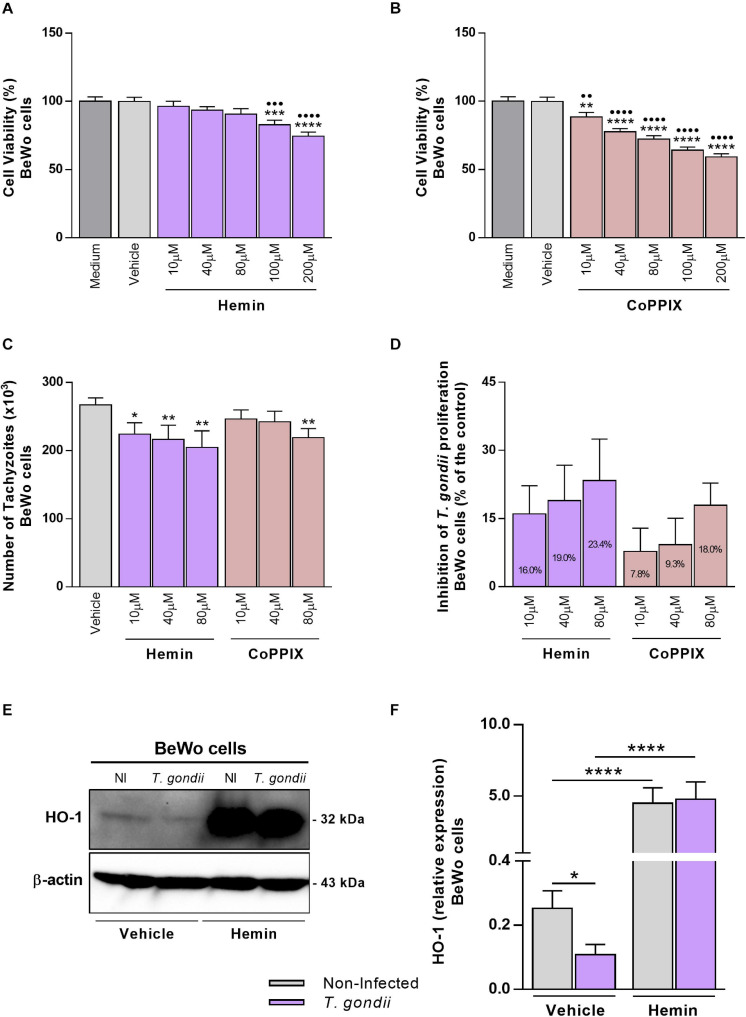
Influence of HO-1 inducers on *T. gondii* proliferation and its expression by BeWo cells. **(A,B)** BeWo (3 × 10^4^ cells/200 μl/well) cells were cultured in 96-well plate during 24 h, treated with hemin or CoPPIX (10, 40, 80, 100, or 200 μM) or incubated with vehicle (NaOH at 0.2%) or only medium (control) for additional 24 h, and submitted to MTT assay. The data were shown as the percentage of viable cells in relation to untreated cells (medium as 100% of viability). Statistical differences in relation to medium (∙) or in relation to vehicle (*). **(C)** BeWo (3 × 10^4^ cells/200 μl/well) cells were cultured for 24 h, infected with *T. gondii* (1:1) for 3 h, treated with hemin or CoPPIX (10, 40, or 80 μM), or incubated with vehicle (NaOH at 0.08%) as control during 24 h, and the *T. gondii* intracellular proliferation was analyzed by the β-galactosidase assay. The data were shown as number of tachyzoites (× 10^3^), used to calculate the inhibition of *T. gondii* proliferation (%) **(D)**. Statistical differences in relation to vehicle (*). **(E,F)** BeWo (1 × 10^6^ cells/2,000 μl/well) cells were cultured for 24 h, infected with *T. gondii* (1:1) or not, as the control of non-infected (NI) cells, for 3 h, treated with hemin (80 μM) or not (NaOH at 0.08% as vehicle) during 24 h, and submitted to western blotting assays for detection of HO-1 and β-actin. The data were shown as representative blots and densitometric analysis for HO-1 relative expression. For all assays, the results were expressed as mean ± SEM from two or three independent experiments performed in six or eight replicates. Significant differences were statistically assessed by one-way ANOVA with Bonferroni’s posttest **(A,B)**, by Kruskal-Wallis with Dunn’s posttest **(C)**, or by Mann-Whitney test **(F)**. ^∗^*P* < 0.05; ^∙∙∗∗^*P* < 0.01; ^∙∙∙∗∗∗^*P* < 0.001; ^∙∙∙∙∗∗∗∗^*P* < 0.0001.

As *T. gondii* infection decreased the HO-1 expression in BeWo cells, we speculate that the induction of HO-1 functional activity could modulate the *T. gondii* proliferation in this trophoblast model. At first, BeWo cells were treated with increasing concentrations of hemin or CoPPIX, the specific chemical inducers of HO-1, and the cellular viability was analyzed by MTT assay. After 24 h of treatments, it was verified that hemin did not alter the viability of BeWo cells at 10, 40, or 80 μM, and doses of 100 μM (*P* = 0.0008) or 200 μM (*P* < 0.0001) reduced significantly the viability in relation to control conditions, medium, or vehicle. Despite this, the cells preserved its viability in 82.8% at 100 μM and 74.5% at 200 μM of hemin treatment (Figure 2A). On the other hand, when BeWo cells were treated with CoPPIX for 24 h, all tested concentrations induced a significant cytotoxicity in a dose-dependent manner, starting with 10 μM, in comparison with medium (*P* = 0.0049) or vehicle (*P* = 0.0052), that was increased with doses of 40, 80, 100, or 200 μM in relation to both control conditions (*P* < 0.0001). The rates of viable cells under CoPPIX-treatment were 88.5, 77.7, 72.2, 64.2, and 59.2% for 10, 40, 80, 100, and 200 μM, respectively (Figure 2B). As doses of 10, 40, and 80 μM of hemin or CoPPIX did not alter or alter in low levels, respectively, the cellular viability of BeWo cells, these concentrations were used to test the effect of this HO-1 inducer on *T. gondii* proliferation.

Next, to evaluate whether the HO-1 induction could affect *T. gondii* intracellular proliferation, BeWo cells were infected with tachyzoites (2F1 clone), treated with hemin or CoPPIX during 24 h, and analyzed for *T. gondii* replication by the β-galactosidase assay. We found that BeWo cells were able to decrease significantly the parasite proliferation when treated with hemin at 10 μM (*P* = 0.0212), 40 μM (*P* = 0.0070), or 80 μM (*P* = 0.0030) or with CoPPIX at 80 μM (*P* = 0.0099) in comparison with vehicle (Figure 2C). In addition, based on data from this assay, we calculated the percentage of the *T. gondii*-growth inhibition for both HO-1 inducers. The results showed that hemin at 10, 40, and 80 μM induced an inhibition of 16, 19, and 23.4%, respectively, on parasite proliferation, and regarding CoPPIX treatments, the same doses inhibited 7.8, 9.3, and 18%, respectively, the tachyzoites replication (Figure 2D). Thus, these findings show that the treatment with HO-1 inducers triggered mechanisms to control *T. gondii* proliferation in BeWo trophoblast cells. Since the hemin stimuli were more effective to inhibit the parasite proliferation in BeWo cells, in relation to CoPPIX, we decided to continue the next assays using hemin at 80 μM for HO-1 induction, as this dose was more effective in controlling the parasite growth.

In addition, in order to verify the effective role of hemin in the induction of HO-1 by *T. gondii*, the enzyme expression of infected BeWo cells was evaluated after treatment with porphyrin. Our data obtained from western blotting analysis reinforce that, in the absence of hemin, BeWo cells decreased significantly the HO-1 levels under *T. gondii* infection in relation to non-infected cells (*P* = 0.0410) (Figures 2E,F), as initially observed. When treated with hemin, *T. gondii*-infected or non-infected BeWo cells increased significantly the HO-1 expression levels in comparison with their respective controls of untreated cells (*P* < 0.0001) (Figures 2E,F), demonstrating the effective capacity of hemin to induce the HO-1 expression. Interestingly, *T. gondii* infection was unable to decrease the HO-1 expression in hemin-treated BeWo cells (Figures 2E,F), in contrast to that observed in infected and untreated cells, highlighting a possible protective role for HO-1, in the control of *T. gondii* proliferation by BeWo trophoblast cells.

### Exogenous Bilirubin Treatment Controls *T. gondii* Proliferation in BeWo Trophoblast Cells

**FIGURE 3:**
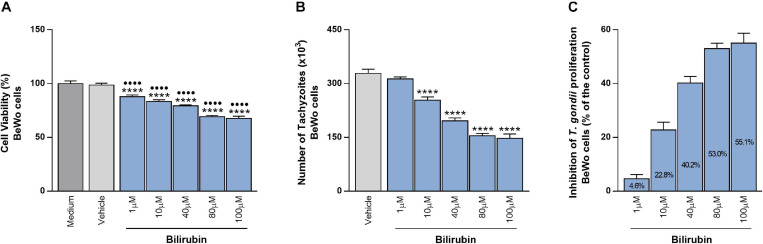
Effects of exogenous bilirubin treatments on *T. gondii* proliferation in BeWo cells. **(A)** BeWo (3 × 10^4^ cells/200 μl/well) cells were cultured in 96-well plate during 24 h, treated with exogenous bilirubin (1, 10, 40, 80, or 100 μM) or incubated with vehicle (DMSO at 0.2%) or only medium (control) for additional 24 h and submitted to MTT assay. The data were shown as the percentage of viable cells in relation to untreated cells (medium as 100% of viability). Statistical differences in relation to medium (∙) or in relation to vehicle (*). **(B)** BeWo (3 × 10^4^ cells/200 μl/well) cells were cultured for 24 h, infected with *T. gondii* (1:1) for 3 h, treated with exogenous bilirubin (1, 10, 40, 80, or 100 μM), or incubated with vehicle (DMSO at 0.2%) during 24 h, and the *T. gondii* intracellular proliferation was analyzed by the β-galactosidase assay. The data were shown as number of tachyzoites (× 10^3^), used to calculate the inhibition of *T. gondii* proliferation (%) **(C)**. Statistical differences in relation to vehicle (*). For all assays, the results were expressed as mean ± SEM from two independent experiments performed in six or eight replicates. Significant differences were statistically assessed by one-way ANOVA with Bonferroni’s posttest **(A,B)**; ^∙∙∙∙∗∗∗∗^*P* < 0.0001.

In order to reinforce the possible beneficial function of HO-1 in the control of *T. gondii* infection, BeWo cells were treated with increasing concentrations of exogenous bilirubin, as an end-product of heme degradation by the HO-1 pathway. At first, the effect of Bilirubin treatment was analyzed in the cellular viability using the MTT assay. After 24 h of treatments, all tested doses of bilirubin reduced significantly the viability of BeWo cells, in a dose-dependent manner, when compared with the control conditions, medium, or vehicle (*P* < 0.0001). Despite this, the cells preserved their viability in 88, 83.4, 79.4, 69.3, and 67.7% at 1, 10, 40, 80, and 100 μM of bilirubin, respectively (Figure 3A). Next, the effect of exogenous bilirubin was evaluated in the *T. gondii* growth and, for this purpose, BeWo cells were infected with tachyzoites, treated with bilirubin at the same concentrations employed in the cellular viability assay, and analyzed for *T. gondii* replication. Data from the β-galactosidase assay demonstrated that, even with the impact on cell viability, the treatments with bilirubin were able to decrease significantly the parasite proliferation from 10 up to 100 μM (*P* < 0.0001), in a dose-dependent manner, when compared with the vehicle condition (Figure 3B). In addition, based on data from this assay, we calculated the percentage of the *T. gondii*-growth inhibition under exogenous bilirubin treatments, and the results showed that bilirubin at 1, 10, 40, 80, and 100 μM induced an inhibition of 4.6, 22.8, 40.2, 53, and 55.1%, respectively, on parasite proliferation (Figure 3C). Thus, based on these findings, we reinforce our hypothesis about the protective role displayed by the HO-1 pathway, since BeWo cells exposed to hemin or bilirubin treatments were less susceptible to *T. gondii* infection.

### *T. gondii* Infection Decreases the p38 MAPK–Phosphorylation in BeWo Cells, but HO-1 Induction Triggers and Sustain Its Activation

**FIGURE 4:**
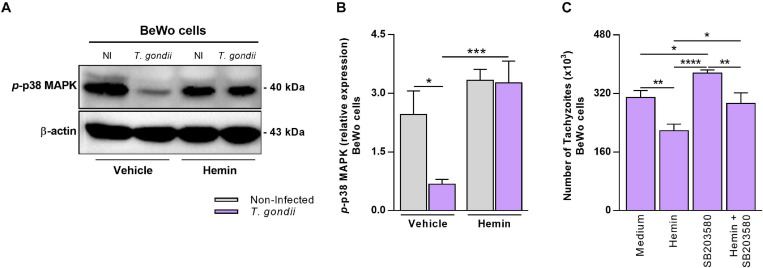
Influence of HO-1 induction on p38 MAPK activation in BeWo cells under *T. gondii* infection. **(A,B)** BeWo (1 × 10^6^ cells/2,000 μl/well) cells were cultured in six-well plate during 24 h, infected with *T. gondii* (1:1) or not (control) for 3 h, treated with hemin (80 μM) or not (NaOH at 0.08% as vehicle) during 24 h, and submitted to western blotting assays for detection of phosphorylated p38 MAPK and β-actin. Protein bands were photodocumented, and densitometric analysis was performed by the ratio among phosphorylated p38 MAPK (*p*-p38 MAPK) and its corresponding β-actin (endogenous control). **(A)** Representative blots for *p*-p38 MAPK and β-actin in the non-infected (NI) or infected (*T. gondii*) and hemin-treated or not (vehicle) BeWo cells. **(B)** Densitometric analysis for *p*-p38 MAPK. **(C)** BeWo (3 × 10^4^ cells/200 μl/well) cells were cultured in 96-well plate during 24 h, pretreated with 10 μM of SB203580 (p38 MAPK inhibitor) or not (control) for 3 h, infected with *T. gondii* (1:1) for 3 h, and treated with hemin (80 μM) or not (control) during 24 h, and the *T. gondii* intracellular proliferation was analyzed by the β-galactosidase assay. The data were shown as number of tachyzoites (× 10^3^). For both assays, the results were expressed as mean ± SEM from two or three independent experiments performed in six or eight replicates. Significant differences were statistically assessed by the Mann-Whitney test (B) or by one-way ANOVA with Bonferroni’s posttest **(C)**; *P < 0.05; **P < 0.01; ***P < 0.001; ****P < 0.0001.

Previous studies reported evidences that the p38 MAPK signaling pathway is commonly related to HO-1 activity, since the stimuli that trigger its induction frequently activate this MAPK in different biological contexts (reviewed by Ryter et al., 2006). Thus, in order to investigate whether *T. gondii* infection could interfere in the p38 MAPK activation in BeWo trophoblast cells and whether the HO-1 induction could be related with p38 MAPK activation in these cells, under parasite infection, we performed western blotting to detect and measure the expression of phosphorylated p38 MAPK (*p*-p38 MAPK). Our data demonstrated that in BeWo cells, *T. gondii* infection reduced significantly the *p*-p38 MAPK levels in comparison with non-infected cells (*P* = 0.0409) (Figures 4A,B). When *T. gondii*-infected BeWo cells were treated with hemin for HO-1 induction, we verified that the *p*-p38 MAPK levels were significantly increased in comparison with the infected cells in the absence of hemin (*P* = 0.0007) (Figures 4A,B). The *p*-p38 MAPK expression under hemin treatment was similar between infected and non-infected cells, demonstrating that in this condition *T. gondii* was unable to decrease the p38 MAPK phosphorylation, as well as in untreated BeWo cells (Figures 4A,B). Additionally, the hemin treatment did not alter the p38 MAPK phosphorylation in non-infected cells (Figures 4A,B). To verify the beneficial effect of the *p*-p38 MAPK in the control of *T. gondii* growth and the HO-1 involvement in this effect, we performed β-galactosidase assays treating cells with the p38 MAPK inhibitor (SB203580) in the presence or absence of hemin and evaluated *T. gondii* proliferation. The data obtained reveal that, as expected, hemin treatment for HO-1 induction decreased significantly the parasite replication in comparison with the medium control condition (*P* = 0.0028), and in addition, the blocking of p38 MAPK activation in the cells via pretreatment with SB203580 favored *T. gondii* proliferation, in relation to medium (*P* = 0.0184) and when compared with hemin-treated cells (*P* < 0.0001) (Figure 4C). Interestingly, when BeWo cells previously inhibited for p38 MAPK activation were treated for HO-1 induction (hemin + SB203580 condition), the *T. gondii* proliferation decreased in relation to SB202580-pretreated cells (*P* = 0.0057), but it was less effective in comparison with the hemin-treated BeWo cells, in the absence of p38 MAPK inhibitor (*P* = 0.0139) (Figure 4C). Based on these findings, we can suggest a possible axis between HO-1 induction and p38 MAPK activation to control *T. gondii* infection in BeWo cells.

### HO-1 Induction Upregulates IL-6 Production in *T. gondii*-Infected BeWo Trophoblast Cells

**FIGURE 5:**
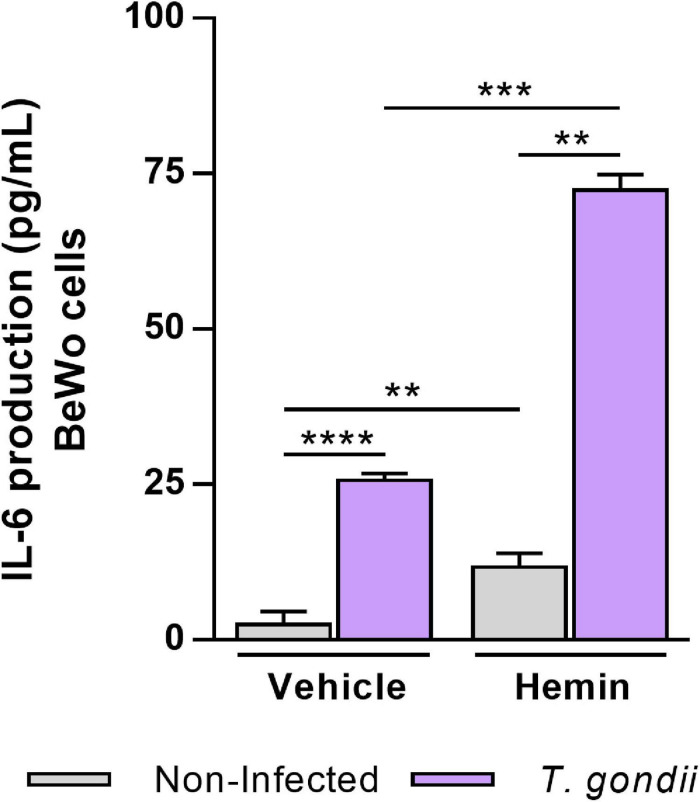
Influence of HO-1 induction on IL-6 production by BeWo cells infected with *T. gondii*. Cell-free supernatants were collected from experiments carried out with BeWo cells infected by *T. gondii* and/or treated with hemin (80 μM) and submitted to cytokine measurement by sandwich ELISA assay. Supernatants from non-infected and/or untreated cells were used as control. The levels of IL-6 secreted by BeWo cells were expressed as mean ± SEM from three independent experiments performed in six or eight replicates. The levels were measured in picograms per milliliter, and the detection limit for IL-6 was 4.7 pg/ml. Significant differences were statistically assessed by the Mann-Whitney test; **P < 0.01; ***P < 0.001; ****P < 0.0001.

Previously, it was demonstrated that BeWo cells modulate its cytokine profile in response to *T. gondii* infection, and particularly when stimulated with IL-6, TNF, and MIF, these trophoblast cells were able to control *T. gondii* proliferation (Barbosa et al., 2014, 2015). In order to investigate whether the HO-1 induction could influence the production of these cytokines favoring the control of *T. gondii* proliferation, we measured the levels of IL-6, TNF, and MIF by the ELISA assay in the supernatants of BeWo cells. In relation to IL-6 production (Figure 5), *T. gondii* infection increased the cytokine secretion by BeWo cells (vehicle condition) in relation to non-infected cells (*P* < 0.0001). Interestingly, in the absence of infection, we observed that the cells induced for HO-1 expression by hemin treatment presented a significant increase on IL-6 levels in comparison with the untreated cells (*P* = 0.0080). When induced for HO-1 (hemin), *T. gondii*-infected BeWo cells produced an increase in IL-6 levels in relation to non-infected and treated cells (*P* = 0.0022), being that this hemin-dependent upregulation was 2.8-fold more pronounced than those observed in *T. gondii*-infected untreated cells (*P* = 0.0001). Thus, these findings suggest that IL-6 may be involved in the mechanisms triggered by HO-1 to control *T. gondii* proliferation in BeWo trophoblast cells. The production of TNF and MIF were also assessed, and the levels of TNF were below the detection limit for this cytokine, whereas the secretion of MIF remained unchanged in any experimental condition of HO-1 induction (data not shown).

### Mechanisms Triggered by the HO-1 Induction to Control *T. gondii* Infection in BeWo Cells

**FIGURE 6:**
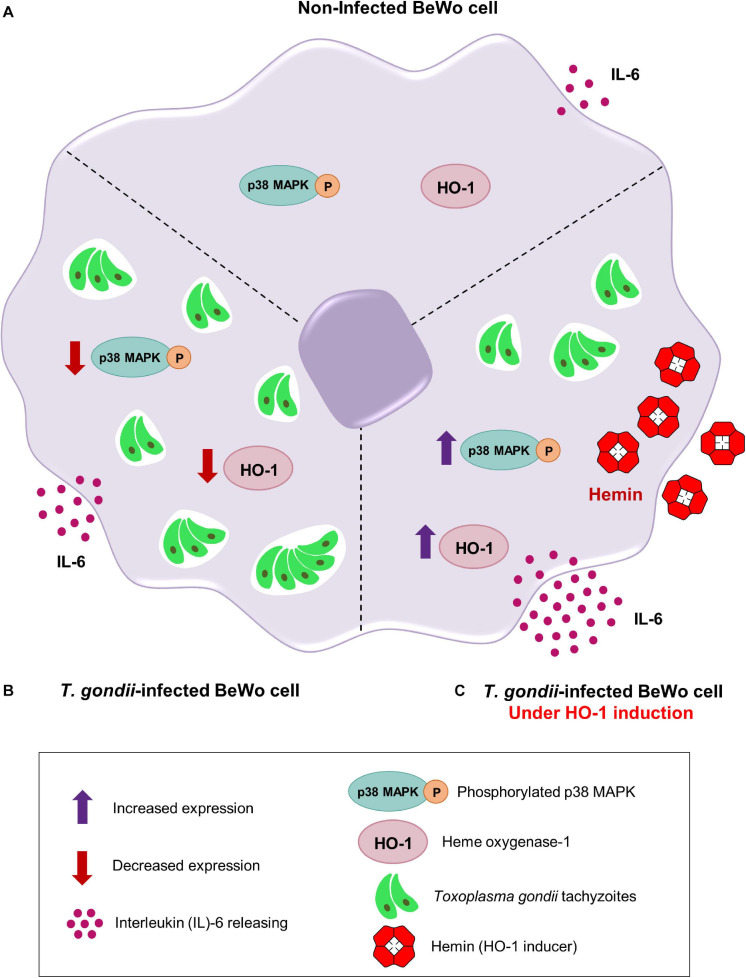
Proposed mechanisms triggered by the HO-1 to control *T. gondii* infection by BeWo trophoblast cells. **(A)** Upper panel, a non-infected BeWo cell expresses normal levels of HO-1 and phosphorylated p38 MAPK, in addition to secreting baseline levels of IL-6. **(B)** Left panel, when BeWo cells were infected with *T. gondii*, the amounts of HO-1 and phosphorylated p38 MAPK were significantly decreased and the IL-6 levels were upregulated. **(C)** Right panel, when finally *T. gondii*-infected BeWo cells were treated with hemin, a specific HO-1 inducer, the parasite proliferation was significantly reduced, sequentially associated with an overexpression of HO-1, an increasing of the p38 MAPK phosphorylation and a releasing of high IL-6 levels, which was higher than those naturally secreted by the *T. gondii*-infected cells non-induced for HO-1 (left panel).

Collectively, our results demonstrated the potential protective role of HO-1 enzyme in the control of *T. gondii* infection in BeWo trophoblast cells, and to clarify the understanding about our findings, we proposed a schematic model (Figure 6). In Figure 6A (upper panel), the represented non-infected BeWo cells express HO-1 and phosphorylated p38 MAPK, in addition to secretion of IL-6 at baseline levels. In Figure 6B (left panel), when BeWo cells were infected with *T. gondii*, the HO-1 expression and the p38 MAPK phosphorylation were significantly decreased, and the IL-6 levels were upregulated in response to infection. Finally, in Figure 6C (right panel), when *T. gondii*-infected BeWo cells were treated with hemin, as HO-1 inducer, the parasite proliferation was significantly decreased in association with an overexpression of HO-1, p38 MAPK phosphorylation, and a releasing of IL-6 at high levels, which was higher than those secreted by the *T. gondii*-infected cells non-treated with hemin.

## Discussion

Impaired trophoblast functions may include impaired trophoblast proliferation, migration, and invasion in addition to impaired cytokine production, which are correlated with human recurrent miscarriages and preeclampsia (Tian et al., 2018; Mo et al., 2019). Over the years, the HO-1 enzyme and the cellular processes linked to its activity have been extensively investigated (Tenhunen et al., 1968, 1969; reviewed by Soares and Bach, 2009). The HO-1 functions are closely associated with inflammation and stress conditions, being that when induced, its activity triggers beneficial mechanisms which characterize it as an important cytoprotective molecule (reviewed by Ryter et al., 2006). During pregnancy, HO-1 exerts essential roles to favor a healthy fetal development, acting in processes such as embryo implantation, placentation, angiogenesis, maternal immune-tolerance and, especially, in the physiology and survival of the trophoblast cells (reviewed by Schumacher and Zenclussen, 2015; Zenclussen et al., 2015). Additionally, HO-1 is expressed by distinct subtypes of trophoblast cells throughout pregnancy, including the villous and extravillous trophoblasts, which play relevant functions at the maternal-fetal interface (Bilban et al., 2009). In parallel, these same trophoblasts are the gateway for pathogens causing infectious diseases, such as *T. gondii*, which infects these placental cells and can lead to congenital toxoplasmosis in the embryo/fetus, after transposing the placental barrier (reviewed by Arora et al., 2017; Heerema-McKenney, 2018). Previously, the HO-1 induction was involved in the control of *T. gondii* infection in animal experimental model (Araujo et al., 2013); however, there is no knowledge about the role of HO-1 during the infection of human trophoblast cells by *T. gondii*. Thus, here, we investigated the influence of the HO-1 enzyme, and cellular mechanisms linked to its induction, in the infection of well-established human trophoblast cells by this parasite.

Initially, to evaluate the impact of *T. gondii* infection in the HO-1 expression by trophoblast cells, we infected BeWo villous and HTR-8/SVneo extravillous trophoblasts and analyzed the enzyme expression levels in these cell lineages. Our results showed that *T. gondii* infection significantly decreases the HO-1 expression in BeWo cells, although the same was not observed in HTR-8/SVneo cell line. This finding related to downregulation of HO-1 in *T. gondii*-infected BeWo cells is in agreement with a previous study whereby H_2_O_2_-exposed retinal pigment epithelial cells (ARPE-19 cell line) demonstrated a similar reduction in HO-1 levels under *T. gondii* infection (RH strain) (Choi et al., 2011). In addition, our result is reinforced by the investigations of Tachibana et al. (2008, 2011), which also showed a downmodulation of HO-1 expression in mouse trophoblast giant (TG) cells, caused by *B. abortus* and *L. monocytogenes* infections. In contrast to BeWo cells, we evidenced that HO-1 expression in HTR-8/SVneo cells remained unchanged after *T. gondii* infection, and in the absence of the parasite, BeWo cells presented higher levels of HO-1 than HTR-8/SVneo, in the same normal physiological condition. Our findings are in accordance with those observed by Bilban et al. (2009), since their study showed a high HO-1 expression in BeWo and primary human cytotrophoblast (CTB) cells, and none or low levels of the enzyme in HTR-8/SVneo and isolated extravillous trophoblasts (EVT), respectively. Based on these surveys, it is possible to hypothesize that *T. gondii* infection did not downregulate the HO-1 levels in HTR-8/SVneo cells because its expression is naturally very low in this EVT lineage, in contrast to BeWo cell line, a model of CTB susceptible to the parasite (Barbosa et al., 2014, 2015; Almeida et al., 2019). In this sense, we decided to use the BeWo villous trophoblast cells in the present study, to continue investigating the mechanisms associated with the relationship between the HO-1 and *T. gondii* infection.

Currently, it is well known that HO-1 is widely related to infectious diseases, and with regard to host-pathogen interaction, its expression and activity triggers pathways that will be beneficial or adverse, depending on the host conditions and pathogen features (reviewed by Singh et al., 2018). Thus, the next step of our investigation was to verify the influence of HO-1 activity on *T. gondii* proliferation in BeWo trophoblast cells, using the porphyrins hemin or CoPPIX, as chemical inducers for HO-1. BeWo cells were first checked for viability after treatment with the porphyrins, in order to define suitable doses of the drugs prior to the quantification of the parasite burden upon HO-1 induction. It was observed that hemin reduced the viability of BeWo cells only at high concentrations (100 and 200 μM), and CoPPIX interfered in the cellular viability from low to high doses (10–200 μM), in a dose-dependent manner. A previous study using BeWo cells as a model to analyze the syncytialization process on human trophoblast observed that the treatments with Hemin reduced significantly the cell viability, ranging from 5% to a maximum of 68%, which was a result of increased apoptosis rates in cells (Liu et al., 2016). In the present study, it was verified that hemin or CoPPIX treatments did not reduce the viability of BeWo cells at more than 40.8% in any used doses; the difference observed from previous studies is probably because Liu et al. (2016) cultured BeWo cells in a serum-starvation condition before stimulating them with hemin, in contrast to our protocol, in which this cell line was cultured under serum supplementation. Thus, the doses of 10, 40, and 80 μM were chosen to treat BeWo cells to verify their impact on *T. gondii* proliferation, since these doses did not interfere or interfered in low levels in the cellular viability when cells were treated with hemin or CoPPIX, respectively.

When we treated *T. gondii*-infected BeWo cells with HO-1 inducers, it was demonstrated that hemin and CoPPIX were able to decrease *T. gondii* intracellular proliferation, being that the effect of hemin was more pronounced in all used concentrations (10, 40, and 80 μM), in a dose-dependent manner, and CoPPIX at a highest dose (80 μM). These findings are in agreement with our previous *in vivo* study, in which the HO-1 induction by hemin treatment decreased the parasite load in the lung and small intestine of C57BL/6 mice infected with ME49 *T. gondii* strain, whereas the treatment with zinc protoporphyrin-IX (ZnPPIX), a HO-1 inhibitor, increased the pulmonary parasitism in infected C57BL/6 and BALB/c mice (Araujo et al., 2013). Moreover, it was previously observed in monocytes from women with pregnancy-associated malaria (PAM) that the increased expression of HO-1 mRNA expression levels were correlated with a low *Plasmodium falciparum* density and high infant birth weight, demonstrating the protective role of HO-1 enzyme against the malaria during pregnancy (Aubouy et al., 2015). Since it was demonstrated that hemin did not affect directly the *T. gondii* tachyzoites (RH strain), after its pretreatment (Araujo et al., 2013), it can suggest that the HO-1 induction probably triggered beneficial mechanisms in BeWo trophoblast cells that interfered in the parasite growth, rather than acting in the parasite directly. Due to the effect of hemin at 80 μM in inhibiting *T. gondii* infection in BeWo cells more effectively than CoPPIX, we chose hemin at this concentration to continue the next steps of this study. To confirm the hemin effectiveness as HO-1 inducer, western blotting analyzes was performed and it was observed that hemin triggered an overexpression of HO-1 in BeWo cells, regardless of infection. Thus, under HO-1 overexpression by hemin treatment, *T. gondii* was unable to decrease the enzyme expression levels. Given these results, we conclude that mechanisms induced by increasing HO-1 expression with hemin treatment decreases *T. gondii* proliferation in BeWo cells.

To reinforce our findings, we treated BeWo cells with exogenous bilirubin, as an end-product resulting from heme degradation by HO-1 (Tenhunen et al., 1969; reviewed by Ryter et al., 2006), and evaluated its influence on *T. gondii* proliferation. We hypothesized that the treatment of BeWo cells with a product from the HO-1 pathway could decrease the parasite replication. As expected, the treatments with bilirubin controlled effectively the *T. gondii* proliferation in the cells, in a dose-dependent manner. Additionally, *in vivo* experiments demonstrated that the exposure of C57BL/6 mice infected with *Plasmodium berghei* ANKA to carbon monoxide (CO), another end-product from the HO-1 pathway, increased the survival and improved the symptoms of the experimental cerebral malaria (ECM), suggesting a protective property of this product against the ECM pathogenesis (Pamplona et al., 2007). Thus, our data suggest that the bilirubin could be an end-product of HO-1 induction involved in the *T. gondii* proliferation in BeWo cells, although further studies are needed to determine the mechanisms triggered by bilirubin that favor parasite control by these cells.

It has been extensively proposed that the regulatory mechanisms underlying the control of HO-1 expression and activity are closely linked to the recruitment of the MAPK signaling pathway. In this context, the stimuli that leads to HO-1 expression and the effects derived from its activity involves the activation of the p38 MAPK, as an upstream or downstream regulatory pathway, respectively (reviewed by Ryter et al., 2006; Paine et al., 2010). Also, during *T. gondii* infection, it is well known the wide contribution of the MAPK in the host cellular response to the parasite growth, mainly through the manipulation of p38 MAPK, in which host cells in response to infection rapidly activates this MAPK, followed by prompt deactivation (reviewed by Denkers et al., 2004). Based on these surveys, we speculated a possible involvement of the p38 MAPK activation, in the control of *T. gondii* infection due to HO-1 induction in BeWo cells. We observed that *T. gondii* infection decreased the levels of phosphorylated p38 MAPK (*p*-p38 MAPK) in untreated BeWo cells, but interestingly, when the cells were treated with hemin, which controls *T. gondii* proliferation by the upregulation of HO-1, the p38 MAPK phosphorylation was increased and sustained even during *T. gondii* infection. Our data are in agreement with Choi et al. (2011), who demonstrated that RH strain of *T. gondii*, besides reducing HO-1 expression, also suppressed the p38 MAPK activation in H_2_O_2_-exposed ARPE-19 cells. Similar with our results, it was shown that *L. monocytogenes* decreased the HO-1 expression in TG cells and, in early stages of infection, reduced the p38 MAPK phosphorylation in these host cells (Hashino et al., 2015). To reinforce our findings, we blocked the p38 MAPK activation in BeWo cells using a specific inhibitor (SB203580), and then analyzed the *T. gondii* proliferation in the context of HO-1 induction. We observed that the inhibition of p38 MAPK with SB203580, in contrast to hemin, led to an increase in *T. gondii* replication in BeWo cells. Interestingly, when the cells previously blocked for p38 MAPK were treated with hemin to trigger the HO-1 induction, the control of *T. gondii* infection was recovered, but less effectively compared with cells induced for HO-1 expression and the p38 MAPK was not inhibited. Thus, the data presented herein suggest that the upregulation of the p38 MAPK activation by HO-1 induction in BeWo cells are involved in the control of *T. gondii* infection. The present study is the first to show the downregulation of p38 MAPK in BeWo cells caused by *T. gondii* infection. Since it is well known that p38 MAPK is strongly activated by a broad range of stress stimuli (reviewed by Roux and Blenis, 2004), which include those that lead the HO-1 induction (reviewed by Ryter et al., 2006), our findings support a possible signaling axis between HO-1 induction and p38 MAPK activation to control *T. gondii* infection in BeWo trophoblast cells.

The control of *T. gondii* proliferation and the improvement of the pathology due to infection, have been closely linked to IL-6 activity, since it was demonstrated the protective role of IL-6 against toxoplasmic encephalitis, using a model of knockout mice (IL-6^–/–^) (Suzuki et al., 1997). Additionally, it was shown that BeWo trophoblasts (Castro et al., 2013; Barbosa et al., 2014, 2015) and THP-1 cells, a human monocyte cell lineage (Castro et al., 2013), increased the levels of IL-6 in response to *T. gondii* infection (RH strain). In the context of HO-1, it was observed that exogenous IL-6 induced the HO-1 expression in primary human macrophages and in murine macrophage cell lineages (Ricchetti et al., 2004). In addition, when human myeloma cells were treated with hemin, the HO-1 induction upregulated the IL-6 secretion *via* p38 MAPK activation (Wu et al., 2016). Based on this crosstalk between HO-1 and IL-6, we analyzed the production of this cytokine by *T. gondii*-infected BeWo cells, under HO-1 induction. Our results demonstrated an increase in the IL-6 secretion by BeWo cells in response to *T. gondii* infection, as previously reported (Castro et al., 2013; Barbosa et al., 2014, 2015), and the HO-1 induction by hemin treatment also enhanced the release of this cytokine, regardless of infection, as observed in myeloma cells (Wu et al., 2016). Interestingly, when *T. gondii*-infected BeWo cells were treated with hemin for HO-1 induction, the levels of IL-6 were significantly upregulated, being higher than those released only in response to infection. It was previously reported that IL-6 produced by BeWo cells favored the human THP-1 monocytes to control *T. gondii* replication, and their own THP-1 cells secreted IL-6 that was able to control the parasite proliferation itself, as an autocrine mechanism (Castro et al., 2013). Since exogenous IL-6 decreased the *T. gondii* proliferation in BeWo cells (Barbosa et al., 2015), we can speculate here that the IL-6 produced by these cells in response to HO-1 induction, could be an autocrine feedback mechanism to control *T. gondii* infection. Future investigations are needed to confirm this hypothesis, as well as to elucidate the exact mechanism underlying the HO-1/IL-6 axis in the control of *T. gondii* proliferation in BeWo trophoblast cells. Taken together, our findings show that the HO-1 induction controls *T. gondii* proliferation in BeWo cells through an upregulation of the p38 MAPK signaling pathway and increased IL-6 production. Herein, we reported for the first time the beneficial role of HO-1 enzyme in the protection of human trophoblast cells against *T. gondii* infection, and also contribute with new data in the development of strategies to control the *T. gondii* vertical transmission.

## Data Availability Statement

The original contributions presented in the study are included in the article/supplementary material, further inquiries can be directed to the corresponding author/s.

## Author Contributions

MA designed and performed the experiments, analyzed and interpreted the results, prepared the figures, and wrote the manuscript. CM contributed in the western blotting analysis. TM and EF contributed with the experimental design and with analytic tools. BB contributed in the interpretation of results and reviewed the manuscript. NS conceived the idea, designed the experiments, interpreted the results, and critically reviewed the manuscript. All authors approved the final version of the manuscript.

## Conflict of Interest

The authors declare that the research was conducted in the absence of any commercial or financial relationships that could be construed as a potential conflict of interest.
